# On the potential of acarbose to reduce cardiovascular disease

**DOI:** 10.1186/1475-2840-13-81

**Published:** 2014-04-16

**Authors:** Eberhard Standl, Michael J Theodorakis, Michael Erbach, Oliver Schnell, Jaakko Tuomilehto

**Affiliations:** 1 Munich Diabetes Research Group e.V. at Helmholtz Centre, Ingolstaedter Landstrasse 1, 85764 Munich, Neuherberg, Germany; 2 Diabetes Trials Unit, University of Oxford, Oxford, UK; 3 Munich Diabetes Research Group e.V. at Helmholtz Centre, Munich, Neuherberg, Germany; 4 Centre for Vascular Prevention, Danube-University Krems, Krems, Austria; 5 Diabetes Prevention Unit, National Institute for Health and Welfare, Helsinki, Finland; 6 Instituto de Investigacion Sanitaria del Hospital Universario LaPaz (IdiPAZ), Madrid, Spain; 7 Diabetes Research Group, King Abdulaziz University, Jeddah, Saudi Arabia

**Keywords:** Acarbose, Cardiovascular, Prevention, Diabetes, Hyperglycaemia, Postprandial

## Abstract

In the emerging landscape of cardiovascular (CV) outcome trials evaluating the effects of blood glucose lowering drugs in individuals with type 2 diabetes, it is becoming increasingly apparent that since the promising signals coming from the United Kingdom Prospective Diabetes Study (UKPDS) no unequivocal benefits have been established for any single therapy thus far. There is an unmet need for introducing an effective pharmacological agent which could target both correlates of glycaemic regulation and CV risk factors, to ameliorate the enormous burden of fatal and non-fatal CV events in diabetic patients. Acarbose, like other alpha-glucosidase inhibitors (AGIs), has been proven to be an effective antidiabetic treatment for decades, but the overall significant impact of this class of drugs on modulating CV risk has only recently been appreciated. Accumulating evidence has shown that apart from its multiple effects on primarily postprandial glucose dysmetabolism, a key component of mechanisms linked to increased incidence of CV events, acarbose therapy also associates with a favorable impact on an array of surrogate markers of CV disease. Data stemming from in vitro testing of human cell lines as well as from preliminary trials in diabetic populations, like the Study to Prevent Non-Insulin-Dependent Diabetes Mellitus (STOP-NIDDM) trial, have highlighted – though not undisputed – the potential beneficial effects of the drug on CV morbidity. Large scale trials, like the ongoing Acarbose Cardiovascular Evaluation (ACE) trial, aim at conclusively establishing such a positive effect in patients with coronary heart disease and impaired glucose tolerance. In view of its usually acceptable level of side effects that are, if they occur, mostly limited to transient gastrointestinal symptoms, acarbose could well be a strong future player in CV disease secondary prevention. Current discouraging results from many trials of antidiabetic medications to significantly lower CV event rates in diabetic patients, should only draw further attention on alternative glucose lowering agents, among which acarbose is indeed promising.

## Introduction

Unexpected – even adverse – cardiovascular (CV) outcome results of recent randomized controlled trials (RCTs) evaluating long term blood glucose lowering therapy in large patient cohorts with type 2 diabetes have gained worldwide attention [1,2]. In particular, the Action to Control Cardiovascular Risk in Diabetes (ACCORD) trial, showing excessive all cause and CV mortality on more intensive glucose lowering therapy, has highlighted the complexity of pros and cons in relation to diabetes treatment [3]. In addition, normalization of glycaemia with HbA1c values within the normal range by applying long acting insulin analogue glargine over six years in the Outcome Reduction with an Initial Glargine Intervention (ORIGIN) trial, was not superior in terms of CV outcomes to more conventional therapy [4]. The latter was based mainly on metformin and/or sulphonylurea therapy and yielded a 0.3% higher mean HbA1c value [4]. Conversely, the CV benefits seen with metformin therapy in a subset of overweight (>120% ideal boody weight) patients in the United Kingdom Prospecitve Diabetes Study (UKPDS, [5]), seem to be largely confined to the comparison without other antidiabetic drug treatment only or to younger patients, according to a recent meta-analysis including 35 RCTs [6]. Finally, saxagliptin and alogliptin, as representatives of the innovative class of dipeptidyl-peptidase-4 (DPP-4) inhibitors have recently failed to demonstrate significant CV outcome benefits in the Saxagliptin Assessment of Vascular Outcomes Recorded in Patients with Diabetes Mellitus (SAVOR-TIMI 53) and the Examination of Cardiovascular Outcomes with Alogliptin versus Standard of Care (EXAMINE) trial [7,8], respectively.

Likewise, the Aleglitazar to reduce Cardiovascular Risk in Coronary Heart Disease (CHD) Patients With a Recent Acute Coronary Syndrome (ACS) Event and Type 2 Diabetes Mellitus (ALECARDIO) trial evaluating the investigational diabetes drug aleglitazar, a dual PPAR alpha/gamma agonist, has been halted due to safety concerns [9]. These were related to bone fractures, heart failure and gastrointestinal bleeding, whereas the intended primary reduction of CV endpoints could not be substantiated. Hence, the effectiveness of blood glucose lowering therapies has only been proven for reducing microvascular complications of diabetes, but has remained controversial, rather disappointing, as to the measurable reduction of macrovascular, i.e. CV disease. In fact, several unwanted potential downsides of some drugs cannot be excluded in this latter context [10]. All blood glucose lowering drugs, therefore, ought to be subjected to RCTs evaluating how they perform in relation to CV complications, even if such studies are not imposed by regulatory authorities. In this paper, we aim to explore the potential of the alpha-glucosidase inhibitor acarbose to reduce CV complications. Acarbose is currently studied in the Acarbose Cardiovascular Evaluation (ACE) Trial [11], in individuals with established coronary heart disease and impaired glucose tolerance, but its results are expected only to become available in about four years.

### Update on mode of action

Recent review papers on acarbose have nicely summarized the mode of action of this drug [11-14]; see also list “Acarbose Highlights of Practical Use” below. In brief, acarbose is a pseudo-carbohydrate that competitively and reversibly inhibits alpha-glucosidases, i.e. membrane-bound intestinal enzymes located in the brush-border of the small intestine mucosa that process oligosaccharides, trisaccharides, and disaccharides to glucose and other monosaccharides. Inhibition of this glucoside hydrolase activity by acarbose delays hydrolysis and digestion of complex carbohydrates in the upper small bowel, subsequently retards absorption of glucose and ‘blunts’ postprandial hyperglycemia. In addition, acarbose exerts the same degree of non-reversible blockade on pancreatic alpha-amylase, which hydrolyzes complex starches to oligosaccharides in the lumen of the small intestine. Intact acarbose is poorly absorbed and is excreted in the feces, mostly intact but with up to 30% undergoing metabolism predominantly via fermentation by colonic microbiota [12].

### Acarbose highlights of practical use

• Acarbose

• Has shown efficacy on the full spectrum of diurnal dysglycemia.

• Is the only oral antidiabetic drug approved in many countries for use in IGT, besides its approval in the treatment of diabetes.

• Provides sustainable glucose control.

• Lowers postprandial hyperglycemia and improves glycemic control.

• Can be combined with other oral antidiabetic drugs and with insulin.

• Has mild to moderate GI side-effects that may be minimized by a stepwise dose regimen.

• Has been shown to be more effective in Asian than in Western populations.

Adaptive changes in the lower smaller intestine, which may take several weeks to occur following initiation of therapy with alpha-glucosidase inhibitors (AGIs), ensure full carbohydrate absorption, mitigate initial gastrointestinal side effects, such as abdominal distension, flatulence or diarrhea and also seem to affect the release of a variety of gastrointestinal hormones, including stimulating secretion of the key incretin hormone glucagon-like peptide-1 (GLP-1, see below). In view of this physiological adaptation process and not infrequent – albeit transient – gastrointestinal side effects, it is advisable to pursue a dosing escalation concept of ‘start low, go slow’. A scheme of 50 to 100 mg tid is usually recommended as effective maintenance therapy.

An indirect consequence of reducing postprandial hyperglycemia with acarbose is an increase of insulin sensitivity. It is thought that by blunting post-ingestion plasma glucose excursions, reduced exposure to glucose toxicity and/or reduced postprandial hyperinsulinemia attenuates down-regulation of the insulin receptor and thus reduces insulin resistance [15]. By directly targeting postprandial hyperglycemia rather than acting as a “conventional” hypoglycemic agent, acarbose is not only aiming at the most inappropriately elevated and longest lasting component of aberrant diurnal glucose metabolism in diabetic individuals, but is also suitable for combination therapy with all other blood glucose lowering agents such as metformin, sulfonylureas, DPP4 inhibitors, GLP-1 agonists, and insulin [11,16].

### Glycemic efficacy

Numerous studies worldwide, have demonstrated the efficacy of acarbose, as monotherapy and combination therapy with other glucose lowering agents, in treating type 2 diabetes [16-27]. In terms of quantifying the glucose-lowering efficacy, a Cochrane systematic [28] review has confirmed a placebo-subtracted decrease of HbA1c with acarbose therapy of 0.8% (95% confidence intervals (CI) 0.64–0.90), a 2.3 mmol/l reduction in postprandial glucose (95% CI 1.92–2.73), and a 1.1 mmol/l reduction in fasting glucose (95% CI 0.83–1.35). In addition, acarbose therapy significantly associates with reduced 1-h post-load insulin concentrations by -40.8 pmol/l [95% CI: -60.6, -21.0] [28].

### Effectiveness in Asian populations

Based on 46 studies, a recent systematic meta-analysis has shown that patients consuming “Eastern” vs. “Western” diets achieve a superior blood glucose lowering effect while on treatment with acarbose [29]. The results revealed that, compared with placebo, mean HbA1c levels were reduced to a significantly greater extent (1.02%) in the Eastern diet group (mean [SD], 1.54% [2.00%]) than in the Western diet group (mean [SD], 0.52% [1.20%]); P < 0.001). The ability of acarbose to reduce HbA1c levels in the Eastern (P = 0.20) and Western (P = 0.10) diet groups was similar to that of sulfonylureas, and HbA1c levels were reduced significantly more (0.39%; P < 0.001) in the Eastern than in the Western diet group. It is not surprising, therefore, that AGIs such as acarbose are much more widely used in Asian countries [11]. Furthermore, it has been recently shown that acarbose is similar to metformin in efficacy in a study in China [30], and is therefore a viable choice for initial therapy in Chinese patients newly diagnosed with type 2 diabetes.

In fact, acarbose has become and still is the most commonly prescribed oral glucose-lowering medication in China with its huge diabetic population [31], where it is also licensed for use in individuals with impaired glucose tolerance, as a means for delaying and or preventing progression to overt type 2 diabetes. Although ethnic specific differences cannot be excluded as an explanation for different glycemic efficacy of acarbose in various populations, merely because of its mechanism of action it seems likely that the starch content, which is still high in most Asian diets, might substantially augment the postprandial hypoglycemic effect of acarbose in these populations and enhance its biopotency [29].

### Effect on gut hormones

There is now firm evidence that all AGIs, including acarbose, increase circulating postprandial active GLP-1 levels and act synergistically to the effect of DPP-4 inhibitors; at the same time, glucose-dependent insulinotropic polypeptide (GIP) is decreased [32-36]. The GLP-1 enhancing effect is particularly seen also in type 2 diabetic patients during standardized meal tests, when administration of AGIs generates significantly lower plasma glucose, serum insulin and total GIP levels, whereas concentrations of active GLP-1, the key incretin hormone, are significantly higher – up to 50% [33]. This notion has been recently reinforced by the finding that 24 weeks of acarbose monotherapy in newly diagnosed patients with T2D was associated with increased levels of both fasting and postprandial GLP-1, NO levels and NOS activity [37]; the benefits of acarbose on cardiovascular risk may, therefore, be related to its stimulation of GLP-1 secretion. Moreover, this effect of acarbose and other AGIs seems to be of additional importance, as clear evidence has been accumulated in recent years that a gradual decline in the incretin effect is a generalized, specific and slowly emerging characteristic in the pathophysiology of type 2 diabetes disease progression [38,39], contrary to earlier beliefs, which linked type 2 diabetes onset with diminished incretin effect [40]. Moreover, combination studies with AGIs on top of treatment with a DPP-4 inhibitor in type 2 diabetic patients, showed further increase of the area under the curve (AUC) of plasma GLP-1 concentrations after a meal, compared to therapy with the DPP-4 inhibitor alone [32,41].

Thus, it seems reasonable to conclude that acarbose and other AGIs might enhance the potentially beneficial effects of GLP-1 on the CV system by increasing GLP-1 levels in a physiological manner [42,43]. At this end, acarbose should also exploit the assumed beneficial CV effects of DPP-4 inhibitors, whose efficacy rely heavily on the functional capacity of the residual beta cell mass to modulate insulin secretion in response to available, slowly dissipating endogenous GLP-1 [38,39].

Apart from its effects on the incretin peptides, acarbose impacts upon release of other gut-derived hormones; it augments the postprandial increase of cholecystokinin (CCK) and peptide YY (PYY) and further potentiates the postprandial reduction of ghrelin, thus suppressing appetite [35]. In conjunction with the prevailing lower postprandial insulin concentrations, these complex effects on entero-endocrine mediators, might impact favorably on the feeling of hunger and regulation of satiety, as well as contribute to less weight gain and even some weight loss while on treatment with acarbose, thus reducing total CV risk further.

Finally, acarbose also mitigates reactive hypoglycemia, e.g. as seen in patients with Roux-en-Y Gastric Bypass operations [44,45].

### Interaction with gut microbiota

One factor in relation to varying glycemic efficacy and perhaps also different incidence and gravity of gastrointestinal side effects, might reside within the enormous and diverse biosphere of the microbiome in the large bowel [46,47] and its capacity to modulate nutrient assimillation. A vast number of microbes with a tenfold higher number of cells compared to the human body colonize the intestine. The so-called microbiota, seem to vary reflecting geographical and cultural differences and have been shown to be associated with incidence of type 2 diabetes, obesity and CV disease [46,47]. Acarbose appears to have a distinct effect on gut microbiota, as it has been shown to increase fecal bifidobacteria content in patients with type 2 diabetes mellitus, thus emphasizing its potential to act in synergy on complex carbohydrate metabolism. This is an intriguing prospect, which warrants further exploration [48].

### Pleiotropic effects

Optimal weight management is a cornerstone of successful diabetes therapy aiming at the prevention of CV complications [1]. Most patients with type 2 diabetes, however, are overweight or obese right at the time of diagnosis and may gain further weight on treatment with drugs like sulphonylureas, glinides, thiazolidindiones and insulin. In contrast, treatment with acarbose has been shown to associate with a moderate weight loss of about 1 kg, on average, compared to placebo, alongside improved glycemic control in most randomized controlled trials [24,49,50]. A recent meta-analysis has confirmed this [51,52], as did a head-to-head comparison with a DPP-4 inhibitor, where acarbose demonstrated significantly greater weight reduction [49]. Furthermore, this advantageous effect on weight management is maintained when the drug is administered in combination with therapeutic options usually leading to weight gain, including new insulin treatment. Administration of acarbose together with initiation of insulin therapy has been found to largely ameliorate the weight gain seen in the placebo control group [24].

Visceral adipose tissue appears to be preferentially targeted in response to treatment with AGIs, e.g. with miglitol [53]. Excessive accumulation of splanchnic fat, however, has also been established as one of the parameters – among other measures of overweight and obesity-related sequelae – most closely linked to CV complications, as evidenced from milestone studies such as DECODE [54], the INTERHEART, the Framingham and the Framingham Heart Offspring Study, the EGIR Group and NAVIGATOR [55-61]. Interestingly, NAVIGATOR showed in some 10,000 subjects with impaired glucose tolerance that waist circumference was one of the strongest predictors of heart failure and stroke [60,61], but also CV complications in general and was superior to measures like weight or body mass index [59]. Also, based on CT scan measurements of visceral fat volume, the Framingham Offspring Heart Study has recently demonstrated a clear superiority of visceral adiposity in predicting CV events as well as cancer, compared to subcutaneous fat mass and BMI [57]. From these findings one might conclude that treatment with AGIs, i.e. miglitol, acarbose, and voglibose, by virtue of better weight management and specific loss of visceral fat, should contribute to net general reduction of CV risk factors (see below) and CV risk.

### Effect on blood pressure

Although not undisputed [62,63], remarkable effects of acarbose on blood pressure and newly diagnosed hypertension have been observed in the randomized controlled STOP-NIDDM trial [64], aiming at preventing type 2 diabetes in subjects with impaired glucose tolerance (IGT). New cases of hypertension were significantly reduced in the acarbose treated group and the mean blood pressure was also significantly lower in this group at the end of the study. A post-hoc planned analysis of the STOP-NIDDM trial also revealed a close correlation of the conversion of IGT to type 2 diabetes and incident cases of hypertension [65]. Likewise, significant reduction of blood pressure was observed in the acarbose-treated patients in the MeRia Study [66], a phenomenon that had been noted also earlier in another randomised controlled trial lasting over 6 months, in comparison with glibenclamide [67]. Although this effect on blood pressure seems important in terms of possible prevention of CV complications, it has not been assessed in a systematic predefined way in most acarbose-related studies and needs further investigation, e.g. in the ongoing ACE trial [11].

In terms of pathophysiology, acarbose has been found to attenuate the blood pressure and splanchnic blood flow response to intraduodenal administration of sucrose in older adults, perhaps due to its multiple effects on the release of intestinal hormones and on insulin secretion [68,69].

### Effects on dyslipidemia

The effectiveness of acarbose to ameliorate postprandial dysmetabolism in patients with diabetes and metabolic syndrome also includes influencing dyslipidemia in a positive manner. Attenuation of postprandial hyperglycemia and hyperinsulinemia associates with reduced triglyceride uptake into adipose tissue, reduced hepatic lipogenesis and hence reduced triglyceride content of the liver [12,70,71]. This is in line also with findings in the STOP-NIDDM trial of slightly, but significantly lower fasting triglycerides and somewhat higher fasting HDL-cholesterol concentrations in the acarbose treated group [64,72]. In addition, acarbose reduces oxidation of LDL-cholesterol [70].

A recent meta-analysis has assessed the effects of DPP-4 inhibitors, pioglitazone, insulin secretagogues, and acarbose on blood lipids when compared to placebo [73]. With respect to triglycerides, a significant reduction could be observed with acarbose, pioglitazone, and DPP-4 inhibitors, but not with sulfonylureas. HDL-C appeared to be increased by treatment with acarbose and pioglitazone, and decreased by sulfonylureas. No effect on total cholesterol was seen with acarbose. So, in aggregate, acarbose affects dyslipidemia favorably, though the effects are not very marked, and its use should be certainly supportive alongside other measures and/or medications to reduce potential CV risk related to dyslipidemia.

### Effect on postprandial platelet activation

The effects of acarbose on platelet activation and its determinants have been recently examined in newly diagnosed, drug-naive type 2 diabetic patients (baseline HbA1c < 7%), as hyperglycemia is a major contributor to in vivo platelet activation in diabetes mellitus [74]. As compared with the placebo group, acarbose-treated patients showed statistically significant reductions in urinary 11-dehydro-TXB2 and 8-iso-PGF2a excretion rate after 8 weeks of treatment (between-group P < 0.001 at 12, 16 and 20 weeks), following earlier decreases in postprandial hyperglycemia and MAGE.

This potentially very important aspect of treatment with acarbose should be generally helpful in reducing CV complications in patients with type 2 diabetes, but has not been fully explored, with the exception of urinary measurements of PGF2a in the context of postprandial hyperglycemia and MAGE (see further below) and of earlier reports on reduction of fibrinogen and prothrombin subsequent to the administration of acarbose [75]. Also, potential interactions with the frequent use of aspirin and other antiplatelet drugs in the context of multifactorial therapy, i.e. the gold standard therapy to manage total CV risk in patients with type 2 diabetes, might be worth studying.

Beyond the effects reducing the challenge of postprandial hyperglycemia, however, acarbose undoubtedly has emerged as an agent also addressing all facets of the known and established metabolic CV risk cluster, including favorable effects on body weight, insulin sensitivity, hyperinsulinemia, blood pressure, dyslipidemia, prothrombotic activation, microbiota-related effects, and gradual diminution of the incretin effect see list “Acarbose Potential to Reduce CVD by Direct Virtue of Its Mode of Action” below.

### Acarbose potential to reduce CVD by direct virtue of its mode of action

• Reduction of postprandial hyperglycemia, glucose variability, and (reactive) hypoglycemia.

• Increased insulin sensitivity.

• Enhanced GLP-1 release.

• Increased postprandial CCK and PYY, reduction of postprandial Ghrelin.

• Impact on and synergy with gut microbiota.

• Induction of moderate weight loss.

• Attenuation of insulin therapy-associated weight gain.

• Decrease of visceral fat.

• Association with lower blood pressure, shift of dyslipidemia to a pattern characterized by more HDL cholesterol and less postprandial triglycerides and oxidized LDL cholesterol.

• Inhibition of key platelet activation pathways.

### Importance of postprandial hyperglycemia and glucose variability on CVD

Postprandial hyperglycemia is a hallmark characteristic in most individuals with impaired glucose tolerance and early type 2 diabetes and has been established as a key pathophysiological component of the mechanism underlying the development of diabetic complications [11]. Fluctuations in glucose levels following a meal are strongly associated with micro- and macrovascular complications not only in patients with type 2 diabetes, but also in individuals with IGT [76]. The risk for developing coronary heart disease or other major adverse CV events is increased by almost three-fold in subjects with IGT compared to people with normal glucose tolerance [77].

Controversy, however, exists as to whether elevated fasting plasma glucose and post-load glucose excursions contribute differently to all-cause mortality or CV outcomes, respectively. A meta-analysis by Coutinho, et al. has suggested that both parameters contribute more or less equally [78], in contrast to other publications, (for example, from the DECODE [54], DECODA [79] or Funagata studies [80]) that support the highly important role of postprandial hyperglycemia contributing to increased CV risk. The A1C-Derived Average Glucose (ADAG) Study, using extensively and repeatedly continuous glucose monitoring to assess the precise relationship between HbA1c concentrations and glycemic parameters, has described a strikingly similar increase of CV risk with all regular glycemic parameters, i.e. with mean blood glucose, fasting blood glucose, 2 hour postprandial blood glucose, and HbA1c in the type 2 diabetic cohort, applying a complex CVD risk factor score [81].

The ongoing prospective AusDiab Study, a cohort study of approximately 10,000 people across Australia having undergone baseline oral glucose tolerance testing, has indicated a graded association between baseline glycemic indices and subsequent CV clinical events and mortality over approximately 5-years of study follow-up, with similar model performance using impaired fasting glucose or IGT classifications [82,83]. The observational CAPRI study in overt diabetic patients treated by diet alone or in combination with oral glucose-lowering drugs, has described an additional relationship between postprandial blood glucose peak values and carotid intima-media thickness, which is above and beyond the association observed with HbA1c [84]. Furthermore, the Diabetes Intervention Study and the San Luigi Gonzaga Study investigators have reported specific associations between postprandial glucose concentrations and risk for subsequent CV events, such as myocardial infarction, in patients with type 2 diabetes [85,86].

A serious limitation of these epidemiological studies is that, most often, postprandial hyperglycemia was not directly measured, but rather inferred by evaluating glycemia after an oral glucose load with 75 g glucose as recommended by international standards [1]. It is noteworthy, therefore, that although the 2-h glucose values after an oral glucose load are closely correlated with the 2-h values after a standardized meal, the correlation with postprandial blood glucose concentrations experienced in the ambulatory setting are much more variable [87]. Continuous glucose monitoring with sensors (CGMS) is probably a necessary prerequisite for studies trying to elucidate the specific impact of postprandial glycemic parameters on CV outcomes, like in the ADAG study [81], especially when it comes to glycemic variability (see also below). This approach has been applied in a number of pathophysiological studies.

Acute increases of blood glucose concentrations and fluctuating glucose levels with high peaks may induce hemodynamic alterations and are associated with endothelial dysfunction and signs of oxidative stress, even in non-diabetic patients. The seminal work of Monnier et al. has shown, by using CGMS in patients with type 2 diabetes, that the urinary excretion of 8-isoprostanes (PGF2a) as a sign of oxidative stress and lipid peroxidation is closely correlated with post-meal glucose levels and even more so with the maximal glucose excursions, in contrast to no such correlation with the HbA1c value or the mean glucose concentration [75]. A similar connection has also been found in the study of Santilli et al, exploring postprandial activation of platelets [74]. Ceriello et al. have used a model of generating glucose fluctuations by glucose infusion both in diabetic and non-diabetic subjects. They found a very close association of the glucose peaks with a decrease in flow-mediated dilatation of the forearm and increased nitrotyrosine, which reflects oxidative stress at mitochondrial level [88].

Finally, Ceriello et al. went on to in vitro studies using human endothelial cells and exposing them to various glucose patterns, i.e. normal or constantly high glucose for 21 days vs. oscillating high and normal glucose for 21 days vs. oscillating high and low or permanently high glucose for 14 days followed by normal glucose by 7 days [89]. They consistently showed that the highest up-regulation of pathways related to apoptosis at the gene transcription level, the highest generation of oxidative stress markers and DNA damage, and the highest reactive oxygen species accumulation were significantly associated with exposure to oscillating glucose concentrations. Interestingly, these alterations could not be restored to normal despite normalization of glucose levels, suggesting a metabolic “memory” of human endothelial cells in response to transient but rapidly changing glycemic milieu [89].

Intervening on postprandial hyperglycemia with several suitable pharmacologic agents, such as short acting insulin analogues, repaglinide, nateglinide and also acarbose has principally proven the reversibility of the pathophysiological CVD surrogate changes discussed above [90-95]. Endothelial function was measured at the myocardial level with positron emission tomography (PET) in insulin-requiring diabetic patients and postprandial hyperglycemia was targeted with various kinds of insulin regimens. When postprandial hyperglycemia was abolished by a rapid acting insulin analogue as compared to regular insulin or placebo, myocardial blood flow was also restored to normal, along with evidence of paradoxical vasoconstriction associated with more marked postprandial hyperglycemia compared to placebo [90]. Similarly, when comparing nateglinide and acarbose to target postprandial hyperglycaemia in new-onset, non-insulin requiring individuals with diabetes, improvement of post-meal flow-mediated dilatation was noted with an edge of acarbose over nateglinde [94] (as to the acarbose studies see further down). Finally, repaglinide has also been shown to reduce CVD surrogates such as CRP, IL-6 and carotid intima media thickness [91].

So, in all, substantial evidence has been accumulated in support of the case that postprandial hyperglycaemia along with fluctuating glucose concentrations with high peaks, provides a pathophysiological link to disordered vasoreactivity in large blood vessels and is a risk predictor for CV complications longer term [77,96,97]. Looking specifically at glucose variability as a CV risk marker using CGMS in epidemiological studies, however, seems to carry some inherent limitations, which could discourage applying this technology on a larger scale. So far, only the Verona Diabetes Study, based on laboratory determinations of FPG, not of postprandial glucose, has published evidence indicating that FPG variability predicted 10 year mortality of type 2 diabetic patients [98]. Conversely, the ADAG study failed to show an increased CV risk associated with CGMS-derived glucose variability parameters in a cross-sectional evaluation both in the type 1 and type 2 diabetic cohorts [81]. Yet shortcomings of this particular study were that CV disease was not looked at prospectively and, in fact, CV disease or events were not assessed at all, but rather a complex CV risk factor score was used instead.

### Efficacy of acarbose to reduce pp hyperglycemia and glucose variability

Numerous studies have demonstrated that acarbose significantly reduces postprandial hyperglycemia, fasting blood glucose and HbA1c levels in patients with type 2 diabetes [11,15,18,23,28,73]. Acarbose also causes significant reductions in postprandial and fasting blood glucose levels in individuals with IGT [23,72]. Also, more recent large scale clinical studies have clearly underlined its high effectiveness in reducing postprandial hyperglycemia and glucose variability, both as mono-therapy or in combination with any other blood glucose-lowering drug, including insulin. More than 15,000 patients have been enrolled in China, Taiwan, the Middle-East, Morocco, Poland, Indonesia, Pakistan and the Philippines and followed up to 3 years [50]. Specific advantages were seen in terms of attenuating postprandial hyperglycaemia, glucose variability, hypoglycaemia and also weight gain. In another study, in which CGMS was used in patients with metformin-treated type 2 diabetes, randomised treatment with acarbose vs. glibenclamide resulted in a much smaller intra-day and inter-day glucose variability in the acarbose group along with a comparable decrease of HbA1c in both treatment groups [99]. This effect was mainly due to a flattening of postprandial hyperglycemia. In addition, several episodes of hypoglycemia occurred with glibenclamide during the 12-week evaluation period, in contrast to none with acarbose.

### Effects of acarbose on CVD surrogates

#### Endothelial dysfunction

Endothelial dysfunction as measured at the forearm or the myocardial blood flow level has been established as a major CVD surrogate predicting serious CV complications, e.g. myocardial infarction. As already noted, acute hyperglycemia within minutes can hamper endothelial function and may even induce paradoxical vasoconstriction. Acarbose unequivocally has been confirmed to prevent such endothelial dysfunction and to restore blood flow to normal in a series of studies examining type 2 diabetic patients in Europe and in Asia [92-94,100]. Moreover, miglitol, another AGI, has also been shown to improve postprandial endothelial dysfunction in patients with acute coronary syndrome and new-onset postprandial hyperglycemia, alluding thus to a potential beneficial class effect [101]. In a head to head comparison, acarbose was superior to nateglinide, a prandial insulin secretagogue, in restoring endothelial dysfunction [94].

#### Microalbuminuria

Microalbuminurea is another clinically important CVD surrogate and highly predictive for cardiovascular morbidity and mortality [1]. Diabetic nephropathy and microalbuminurea have been found linked especially with postprandial hyperglycemia, among other glycemic parameters [102,103], whereas treatment with acarbose and other AGIs not only reduced postprandial hyperglycemia, but also microalbuminurea and related pro-inflammatory factors, such as interleukin 18 [103,104]. In addition, there is a wealth of animal studies proving acarbose to delay or prevent the onset of renal, retinal, lens and neurological changes and the development of ischaemic myocardial lesions [105].

#### Carotid intima-media thickening

Carotid intima-media thickening (CIMT) assessed by Duplex sonography, represents another key CVD risk surrogate [1]. Acarbose and other AGIs have uniformly and consistently been shown to reduce progression of CIMT in randomized controlled trials both in Europe and Asia [106-109]. A recent meta-analysis has strengthened this notion further [110]. However, a recent study has also emphasized that although acarbose delayed progression of CIMT in early diabetes, reductions in glycemia were not major determinants of reduced rate of IMT progression [111]. Vascular benefits of acarbose might be independent of its glycemic effects, therefore.

#### Oxidative stress and low grade inflammation

Oxidative stress and low grade inflammation in man are usually assessed by biomarkers, e.g. urinary excretion of PGF2a or cellular nuclear factor-kappa B (NF-kB) activation or nitrotyrosine, tumor necrosis factor (TNF) alpha, interleukin 6 or high sensitive C-reactive protein (CRP) in serum [77]. In particular the relationship between CRP concentrations and CV events has been studied extensively (1, 104). Marginally elevated CRP levels showed strong predictive capability for CV events in numerous epidemiological longitudinal studies and have also been targeted in large randomized intervention trials, e.g. the JUPITER trial [112]. Reduction of slightly elevated CRP concentrations in an otherwise healthy primary prevention population by rosuvastatin in this trial, was linked to significantly lower proportion of CV events. Thus, biomarkers like high sensitive CRP are today widely used as surrogates for CVD in interventional or observational studies [1].

Against this background, it appears reassuring that acarbose has also been found to reduce established markers of low grade inflammation and oxidative stress, such as serum CRP concentrations in subjects with IGT, and cellular NF-kB activation or urinary PGF2a in type 2 diabetic patients [113,114]. In addition, acarbose reduced the post meal net electronegative charge of LDL in patients with newly diagnosed type 2 diabetes and also the post meal white blood cell response in patients with early type 2 diabetes in the AI (I) DA Study [115,116].

Expanding studies of hypoxic stress in the context of postprandial hyperglycemia-induced pathologic changes into animal models of intermittent hypoxia, acarbose was able to prevent both cardiac interstitial fibrosis and hypertrophy of cardio-myocytes [117]. So, in aggregate, acarbose may show unique effectiveness in favorably influencing established CV surrogates like endothelial dysfunction, CIMT or markers of oxidative stress and low grade inflammation (see list “Acarbose Potential to Reduce CVD by Effects on CVD Surrogate Outcomes” below).

### Acarbose potential to reduce CVD by effects on CVD surrogate outcomes

• Restoration of endothelial function.

• Reduction of microalbuminuria and nephropathy (also animal studies).

• Reduction of carotid intima-media thickening.

• Reduction of oxidative stress.

• Reduction of low grade inflammation.

• Reduction of cardiac interstitial fibrosis (only animal studies).

### Trial evidence of acarbose to reduce CV events

The Study to Prevent Non-Insulin-Dependent Diabetes Mellitus (STOP-NIDDM) Trial evaluated the efficacy of 3 years of treatment with acarbose vs. placebo in preventing the transition to overt diabetes among 1429 patients with IGT [64,72]. Targeting postprandial hyperglycaemia with acarbose was not only found to be associated with a 36% reduction in the incidence of new onset diabetes as the primary objective of this study, but also with a highly significant reduction of myocardial infarction (1 vs. 12, p = 0.0226) and any CV event (15 vs. 32, p = 0.0326), as assessed by a planned post hoc analysis of predefined secondary outcomes. Consistent with these findings, another post hoc meta-analysis of seven randomised trials with acarbose in 2180 patients with type 2 diabetes and a follow-up of 1 year or longer ([66], MeRia Study), likewise revealed a significant decrease of myocardial infarction (9 vs. 19, p = 0.012) and any CV event (76 vs. 88, p = 0.006).

Although these data might be suggestive for a favorable effect of acarbose to prevent CV events, one should keep in mind that the number of events was indeed small and the analysis done retrospectively, thus these results were only hypothesis generating [64,66]. A definitive randomized placebo-controlled trial, however, seems to be warranted to test the hypothesis that acarbose could reduce CV events as a primary outcome in a large enough cohort of high CV risk patients with dysglycemia. Of particular relevance, the latter goes often unrecognized and may be first diagnosed within the context of an acute coronary syndrome [1]. The ACE Trial started in 2009 and was designed to examine the potential of acarbose to reduce CV complications in a secondary prevention population with impaired glucose tolerance [118].

### ACE trial: brief outline of study design

The ACE trial is a randomized, placebo-controlled, double-blind, pragmatic secondary prevention trial, investigator initiated and academically led by the Oxford Trial Unit. It is planned to enroll 7500 patients in China and Hong Kong with IGT and coronary heart disease as defined by a prior myocardial infarction (MI), unstable angina or current stable angina. Patients are randomized to 50 mg acarbose tid or matching placebo in a 1:1 fashion on top of optimized guideline based CV care and at least 3 months out from a prior MI. The primary composite CV outcome is the time to the first occurrence of CV death, nonfatal MI or nonfatal stroke. Secondary outcomes include prevention of diabetes and all-cause mortality.

## Conclusions

### Perspectives and expectations

Recent results of RCTs evaluating blood glucose lowering drugs and looking at longer-term CV outcomes have underlined the importance of performing RCTs. Hopes derived from rather short-term labelling trials and pathophysiological studies assessing CV biomarkers or CV surrogate outcomes may turn out to be erroneous when the longer term outcome results from RCTs become available. As shown in this review, acarbose is a blood glucose lowering drug of great promise considering the compelling evidence for its favorable influence on most CV risk factors and CV surrogates. In the rapidly evolving landscape of CV outcomes trials, this fosters our expectation that the ongoing ACE RCT might eventually confirm the positive post hoc CV results coming from the STOP-NIDDM and the MeRia trials.

## Competing interests

ES, OS, JT received lecturing fees from a number of pharmaceutical companies, including Bayer Health Care. ES, MJT, JT serve on committees of the academically led ACE trial.

## Authors’ contributions

All authors contributed to conception and design, drafting the article, revising the article critically, and final approval of the version to be published.

## References

[B1] Ryden L Grant PJ Anker SD Berne C Cosentino F Danchin N Deaton C Escaned J Hammes HP Huikuri H Marre M Marx N Mellbin L Ostergren J Patrono C Seferovic P Uva MS Taskinen MR Tendera M Tuomilehto J Valensi P Zamorano JL Achenbach S Baumgartner H Bax JJ Bueno H Dean V Erol C Fagard R Ferrari R ESC Guidelines on diabetes, pre-diabetes, and cardiovascular diseases developed in collaboration with the EASD: the Task Force on diabetes, pre-diabetes, and cardiovascular diseases of the European Society of Cardiology (ESC) and developed in collaboration with the European Association for the Study of Diabetes (EASD) Eur Heart J 2013 34 39 3035 3087 23996285 10.1093/eurheartj/eht108

[B2] Inzucchi SE Bergenstal RM Buse JB Diamant M Ferrannini E Nauck M Peters AL Tsapas A Wender R Matthews DR Management of hyperglycaemia in type 2 diabetes: a patient-centered approach. Position statement of the American Diabetes Association (ADA) and the European Association for the Study of Diabetes (EASD) Diabetologia 2012 55 6 1577 1596 10.1007/s00125-012-2534-0 22526604

[B3] Gerstein HC Miller ME Byington RP Goff DC Jr Bigger JT Buse JB Cushman WC Genuth S Ismail-Beigi F Grimm RH Jr Probstfield JL Simons-Morton DG Friedewald WT Effects of intensive glucose lowering in type 2 diabetes N Engl J Med 2008 358 24 2545 2559 18539917 10.1056/NEJMoa0802743 PMC4551392

[B4] Gerstein HC Bosch J Dagenais GR Diaz R Jung H Maggioni AP Pogue J Probstfield J Ramachandran A Riddle MC Rydén LE Yusuf S Basal insulin and cardiovascular and other outcomes in dysglycemia N Engl J Med 2012 367 4 319 328 22686416 10.1056/NEJMoa1203858

[B5] UK Prospective Diabetes Study (UKPDS) Group Effect of intensive blood-glucose control with metformin on complications in overweight patients with type 2 diabetes (UKPDS 34). UK Prospective Diabetes Study (UKPDS) Group Lancet 1998 352 9131 854 865 9742977

[B6] Lamanna C Monami M Marchionni N Mannucci E Effect of metformin on cardiovascular events and mortality: a meta-analysis of randomized clinical trials Diabetes Obes Metab 2011 13 3 221 228 10.1111/j.1463-1326.2010.01349.x 21205121

[B7] Scirica BM Bhatt DL Braunwald E Steg PG Davidson J Hirshberg B Ohman P Frederich R Wiviott SD Hoffman EB Cavender MA Udell JA Desai NR Mosenzon O McGuire DK Ray KK Leiter LA Raz I Saxagliptin and cardiovascular outcomes in patients with type 2 diabetes mellitus N Engl J Med 2013 369 14 1317 1326 10.1056/NEJMoa1307684 23992601

[B8] White WB Cannon CP Heller SR Nissen SE Bergenstal RM Bakris GL Perez AT Fleck PR Mehta CR Kupfer S Wilson C Cushman WC Zannad F Alogliptin after acute coronary syndrome in patients with type 2 diabetes N Engl J Med 2013 369 14 1327 1335 10.1056/NEJMoa1305889 23992602

[B9] Nainggolan L ALECARDIO trial halted for safety. Medscape Cardiology 2013 Available at: http://www.medscape.com/viewarticle/793304?t=1 (accessed 19-02-2014)

[B10] Standl E Saxagliptin, alogliptin, and cardiovascular outcomes N Engl J Med 2014 370 5 483 24476445 10.1056/NEJMc1313880

[B11] Standl E Schnell O Alpha-glucosidase inhibitors 2012 - cardiovascular considerations and trial evaluation Diab Vasc Dis Res 2012 9 3 163 169 10.1177/1479164112441524 22508699

[B12] Bischoff H The mechanism of alpha-glucosidase inhibition in the management of diabetes Clin Invest Med 1995 18 4 303 311 8549017

[B13] Hanefeld M Schaper F Acarbose: oral anti-diabetes drug with additional cardiovascular benefits Expert Rev Cardiovasc Ther 2008 6 2 153 163 10.1586/14779072.6.2.153 18248270

[B14] Rosak C Mertes G Critical evaluation of the role of acarbose in the treatment of diabetes: patient considerations Diabetes Metab Syndr Obes 2012 5 357 367 23093911 10.2147/DMSO.S28340 PMC3476372

[B15] Lebovitz HE Alpha-glucosidase inhibitors as agents in the treatment of diabetes Diab Rev 1998 6 132 145

[B16] Holman RR Cull CA Turner RC A randomized double-blind trial of acarbose in type 2 diabetes shows improved glycemic control over 3 years (U.K. Prospective Diabetes Study 44) Diabetes Care 1999 22 6 960 964 10.2337/diacare.22.6.960 10372249

[B17] Baotic I Profozic V Metelko Z Crncevic-Orlic Z Dvorscak D Grabovac A Jovic-Paskvalin L Kolacio Z Komadina R Misura I Pavlic-Renar I Persic M Petrek-Solic B Potocic D Suznjevic J Svarc Z Benefits of acarbose use in patients with non-insulin-dependent diabetes mellitus Diabetologia Croatia 2000 29 147 153

[B18] Chiasson JL Josse RG Hunt JA Palmason C Rodger NW Ross SA Ryan EA Tan MH Wolever TM The efficacy of acarbose in the treatment of patients with non-insulin-dependent diabetes mellitus. A multicenter controlled clinical trial Ann Intern Med 1994 121 12 928 935 10.7326/0003-4819-121-12-199412150-00004 7734015

[B19] Coniff RF Shapiro JA Seaton TB Hoogwerf BJ Hunt JA A double-blind placebo-controlled trial evaluating the safety and efficacy of acarbose for the treatment of patients with insulin-requiring type II diabetes Diabetes Care 1995 18 7 928 932 10.2337/diacare.18.7.928 7555551

[B20] Costa B Fernández-Álvarez J Fuentes CM Martín F Molto E López E Three-Year Results of Spanish PREDIAP Trial. New Data about Effectiveness of Acarbose for Type 2 Diabetes Prevention Diabetes 2005 54 suppl 1 A608

[B21] Hwu CM Ho LT Fuh MM Siu SC Sutanegara D Piliang S Chan JC Acarbose improves glycemic control in insulin-treated Asian type 2 diabetic patients: results from a multinational, placebo-controlled study Diabetes Res Clin Pract 2003 60 2 111 118 10.1016/S0168-8227(03)00015-9 12706319

[B22] Lindstrom J Tuomilehto J Spengler M Acarbose treatment does not change the habitual diet of patients with Type 2 diabetes mellitus. The Finnish Acargbos Study Group Diabet Med 2000 17 1 20 25 10.1046/j.1464-5491.2000.00210.x 10691155

[B23] Pan CY Landen H Post-marketing surveillance of acarbose treatment in patients with type 2 diabetes mellitus and subjects with impaired glucose tolerance in China Clin Drug Investig 2007 27 6 397 405 10.2165/00044011-200727060-00003 17506590

[B24] Schnell O Mertes G Standl E Acarbose and metabolic control in patients with type 2 diabetes with newly initiated insulin therapy Diabetes Obes Metab 2007 9 6 853 858 10.1111/j.1463-1326.2006.00666.x 17924867

[B25] Segal P Eliahou HE Petzinna D Neuser D Bruckner A Spengler M Long-term efficacy and tolerability of acarbose treatment in patients with type 2 diabetes mellitus Clin Drug Investig 2005 25 9 589 595 10.2165/00044011-200525090-00004 17532703

[B26] Su SO Zhao J Zhang J Zou D Li H Sheng Z Liang GX Landen H Efficacy, safety and acceptance of Acarbose treatment under day-to-day clinical practice conditions: Post-Marketing Surveillance in Chinese type 2 diabetic patients Chin J Endocrinol Metab 2006 22 6a-1 6a-5

[B27] Sumual AR Pandelaki K Rotty LAW Acarbose/Metformin Combination Versus Metformin Alone in Indonesian Patients With Type 2 Diabetes J Asean fed Endocr Societies 2003 21 24 31

[B28] van de Laar FA Lucassen PL Akkermans RP van de Lisdonk EH Rutten GE van Weel C Alpha-glucosidase inhibitors for patients with type 2 diabetes: results from a Cochrane systematic review and meta-analysis Diabetes Care 2005 28 1 154 163 10.2337/diacare.28.1.154 15616251

[B29] Zhu Q Tong Y Wu T Li J Tong N Comparison of the hypoglycemic effect of acarbose monotherapy in patients with type 2 diabetes mellitus consuming an Eastern or Western diet: a systematic meta-analysis Clin Ther 2013 35 6 880 899 10.1016/j.clinthera.2013.03.020 23602502

[B30] Yang W Liu J Shan Z Tian H Zhou Z Ji Q Weng J Jia W Lu J Xu Y Yang Z Chen W Acarbose compared with metformin as initial therapy in patients with newly diagnosed type 2 diabetes: an open-label, non-inferiority randomised trial Lancet Diab Endocrinol 2014 2 1 46 55 10.1016/S2213-8587(13)70021-4 24622668

[B31] IMSPlus CHPA, IMS Global Services China Hospital Pharmaceutical Audit. Quarterly Market Brief 2Q2009 and China Pharmaceutical Market Snapshot Q2/2011 2011 available at http://www.IMSHealth.com (accessed 15-01-2014)

[B32] Aoki K Kamiyama H Yoshimura K Shibuya M Masuda K Terauchi Y Miglitol administered before breakfast increased plasma active glucagon-like peptide-1 (GLP-1) levels after lunch in patients with type 2 diabetes treated with sitagliptin Acta Diabetol 2012 49 3 225 230 10.1007/s00592-011-0322-9 21898126

[B33] Narita T Yokoyama H Yamashita R Sato T Hosoba M Morii T Fujita H Tsukiyama K Yamada Y Comparisons of the effects of 12-week administration of miglitol and voglibose on the responses of plasma incretins after a mixed meal in Japanese type 2 diabetic patients Diabetes Obes Metab 2012 14 3 283 287 10.1111/j.1463-1326.2011.01526.x 22051162

[B34] Qualmann C Nauck MA Holst JJ Orskov C Creutzfeldt W Glucagon-like peptide 1 (7-36 amide) secretion in response to luminal sucrose from the upper and lower gut. A study using alpha-glucosidase inhibition (acarbose) Scand J Gastroenterol 1995 30 9 892 896 10.3109/00365529509101597 8578189

[B35] Enc FY Imeryuz N Akin L Turoglu T Dede F Haklar G Tekesin N Bekiroglu N Yegen BC Rehfeld JF Holst JJ Ulusoy NB Inhibition of gastric emptying by acarbose is correlated with GLP-1 response and accompanied by CCK release Am J Physiol Gastrointest Liver Physiol 2001 281 3 G752 G763 11518688 10.1152/ajpgi.2001.281.3.G752

[B36] Yabe D Watanabe K Sugawara K Kuwata H Kitamoto Y Sugizaki K Fujiwara S Hishizawa M Hyo T Kuwabara K Yokota K Iwasaki M Kitatani N Kurose T Seino Y Effects of sitagliptin, acarbose and sulfonylureas on postprandial levels of GLP-1 and GIP in Japanese patients with type 2 diabetes Diabetologia 2011 54 Suppl1 S342

[B37] Zheng MY Yang JH Shan CY Zhou HT Xu YG Wang Y Ren HZ Chang BC Chen LM Effects of 24-week treatment with acarbose on glucagon-like peptide 1 in newly diagnosed type 2 diabetic patients: a preliminary report Cardiovasc Diabetol 2013 12 73 10.1186/1475-2840-12-73 23642288 PMC3653752

[B38] Holst JJ Knop FK Vilsboll T Krarup T Madsbad S Loss of incretin effect is a specific, important, and early characteristic of type 2 diabetes Diabetes Care 2011 34 Suppl 2 S251 S257 21525464 10.2337/dc11-s227 PMC3632188

[B39] Nauck MA Vardarli I Deacon CF Holst JJ Meier JJ Secretion of glucagon-like peptide-1 (GLP-1) in type 2 diabetes: what is up, what is down? Diabetologia 2011 54 1 10 18 10.1007/s00125-010-1896-4 20871975

[B40] Theodorakis MJ Carlson O Michopoulos S Doyle ME Juhaszova M Petraki K Egan JM Human duodenal enteroendocrine cells: source of both incretin peptides, GLP-1 and GIP Am J Physiol Endocrinol Metab 2006 290 3 E550 E559 16219666 10.1152/ajpendo.00326.2004

[B41] Kusunoki Y Katsuno T Myojin M Miyakoshi K Ikawa T Matsuo T Ochi F Tokuda M Murai K Miuchi M Hamaguchi T Miyagawa J Namba M Effect of additional administration of acarbose on blood glucose fluctuations and postprandial hyperglycemia in patients with type 2 diabetes mellitus under treatment with alogliptin Endocr J 2013 60 4 431 439 23220949

[B42] Lonborg J Vejlstrup N Kelbaek H Botker HE Kim WY Mathiasen AB Jorgensen E Helqvist S Saunamaki K Clemmensen P Holmvang L Thuesen L Krusell LR Jensen JS Kober L Treiman M Holst JJ Engstrom T Exenatide reduces reperfusion injury in patients with ST-segment elevation myocardial infarction Eur Heart J 2012 33 12 1491 1499 10.1093/eurheartj/ehr309 21920963

[B43] Wang D Luo P Wang Y Li W Wang C Sun D Zhang R Su T Ma X Zeng C Wang H Ren J Cao F Glucagon-like peptide-1 protects against cardiac microvascular injury in diabetes via a cAMP/PKA/Rho-dependent mechanism Diabetes 2013 62 5 1697 1708 10.2337/db12-1025 23364453 PMC3636622

[B44] Ritz P Vaurs C Bertrand M Anduze Y Guillaume E Hanaire H Usefulness of acarbose and dietary modifications to limit glycemic variability following Roux-en-Y gastric bypass as assessed by continuous glucose monitoring Diabetes Technol Ther 2012 14 8 736 740 10.1089/dia.2011.0302 22853724

[B45] Buscemi S Mattina A Genova G Genova P Nardi E Costanzo M Seven-day subcutaneous continuous glucose monitoring demonstrates that treatment with acarbose attenuates late dumping syndrome in a woman with gastrectomy for gastric cancer Diabetes Res Clin Pract 2013 99 1 e1 e2 10.1016/j.diabres.2012.10.021 23146372

[B46] Qin J Li Y Cai Z Li S Zhu J Zhang F Liang S Zhang W Guan Y Shen D Peng Y Zhang D Jie Z Wu W Qin Y Xue W Li J Han L Lu D Wu P Dai Y Sun X Li Z Tang A Zhong S Li X Chen W Xu R Wang M Feng Q A metagenome-wide association study of gut microbiota in type 2 diabetes Nature 2012 490 7418 55 60 10.1038/nature11450 23023125

[B47] Karlsson FH Tremaroli V Nookaew I Bergstrom G Behre CJ Fagerberg B Nielsen J Backhed F Gut metagenome in European women with normal, impaired and diabetic glucose control Nature 2013 498 7452 99 103 10.1038/nature12198 23719380

[B48] Liu HX Li J Liu B Liu DD Sunli YJ Zhang P Meng XX Su BL Effect of acarbose on fecal bifidobacteria content in patients with type 2 diabetes mellitus Chin J Endocrinol Metab 2011 27 47 50

[B49] Pan C Yang W Barona JP Wang Y Niggli M Mohideen P Foley JE Comparison of vildagliptin and acarbose monotherapy in patients with Type 2 diabetes: a 24-week, double-blind, randomized trial Diabet Med 2008 25 4 435 441 10.1111/j.1464-5491.2008.02391.x 18341596

[B50] Li C Hung YJ Qamruddin K Aziz MF Stein H Schmidt B International noninterventional study of acarbose treatment in patients with type 2 diabetes mellitus Diabetes Res Clin Pract 2011 92 1 57 64 10.1016/j.diabres.2010.12.033 21251726

[B51] Schnell O Sheu WH Watada H Kalra S Yamamoto N An Alpha-Glucosidase Inhibitor, Acarbose, Reduces Body Weight Irrespective of Glycemic Control Status Diabetes 2013 62 Suppl 1 A553

[B52] Nakhaee A Sanjari M Evaluation of effect of acarbose consumption on weight losing in non-diabetic overweight or obese patients in Kerman J Res Med Sci 2013 18 5 391 394 24174943 PMC3810572

[B53] Shimabukuro M Higa M Yamakawa K Masuzaki H Sata M Miglitol, alpha-glycosidase inhibitor, reduces visceral fat accumulation and cardiovascular risk factors in subjects with the metabolic syndrome: a randomized comparable study Int J Cardiol 2013 167 5 2108 2113 10.1016/j.ijcard.2012.05.109 22721642

[B54] Glucose tolerance and mortality: comparison of WHO and American Diabetes Association diagnostic criteria The DECODE study group. European Diabetes Epidemiology Group. Diabetes Epidemiology: Collaborative analysis Of Diagnostic criteria in Europe Lancet 1999 354 9179 617 621 10466661

[B55] Yusuf S Hawken S Ounpuu S Dans T Avezum A Lanas F McQueen M Budaj A Pais P Varigos J Lisheng L Effect of potentially modifiable risk factors associated with myocardial infarction in 52 countries (the INTERHEART study): case-control study Lancet 2004 364 9438 937 952 10.1016/S0140-6736(04)17018-9 15364185

[B56] Kannel WB Cupples LA Ramaswami R Stokes J 3rd Kreger BE Higgins M Regional obesity and risk of cardiovascular disease; the Framingham Study J Clin Epidemiol 1991 44 2 183 190 10.1016/0895-4356(91)90265-B 1995775

[B57] Britton KA Massaro JM Murabito JM Kreger BE Hoffmann U Fox CS Body fat distribution, incident cardiovascular disease, cancer, and all-cause mortality J Am Coll Cardiol 2013 62 10 921 925 10.1016/j.jacc.2013.06.027 23850922 PMC4142485

[B58] Balkau B Deanfield JE Despres JP Bassand JP Fox KA Smith SC Jr Barter P Tan CE Van Gaal L Wittchen HU Massien C Haffner SM International Day for the Evaluation of Abdominal Obesity (IDEA): a study of waist circumference, cardiovascular disease, and diabetes mellitus in 168,000 primary care patients in 63 countries Circulation 2007 116 17 1942 1951 10.1161/CIRCULATIONAHA.106.676379 17965405 PMC2475527

[B59] Preiss D Thomas LE Sun JL Haffner SM Holman RR Standl E Leiter LA Mazzone T Rutten GE Tognoni G Martinez FA Chiang FT Califf RM McMurray JJ Predictors of cardiovascular events in a contemporary population with impaired glucose tolerance: an observational analysis of the Nateglinide and Valsartan in impaired glucose tolerance outcomes research (NAVIGATOR) trial BMJ Open 2012 2 6 e001925 doi:10.1136/bmjopen-2012-001925 23204139 10.1136/bmjopen-2012-001925 PMC3533049

[B60] Wong YW Thomas L Sun JL McMurray JJ Krum H Hernandez AF Rutten GE Leiter LA Standl E Haffner SM Mazzone T Martinez FA Tognoni G Giles T Califf RM Predictors of incident heart failure hospitalizations among patients with impaired glucose tolerance: insight from the Nateglinide And Valsartan in Impaired Glucose Tolerance Outcomes Research study Circ Heart Fail 2013 6 2 203 210 10.1161/CIRCHEARTFAILURE.112.000086 23388113

[B61] Preiss D Giles TD Thomas LE Sun JL Haffner SM Holman RR Standl E Mazzone T Rutten GE Tognoni G Chiang FT McMurray JJ Califf RM Predictors of stroke in patients with impaired glucose tolerance: results from the Nateglinide and Valsartan in Impaired Glucose Tolerance Outcomes Research trial Stroke 2013 44 9 2590 2593 10.1161/STROKEAHA.113.001177 23899915

[B62] Kaiser T Sawicki PT Acarbose for patients with hypertension and impaired glucose tolerance JAMA 2003 290 23 3066 author reply 3067-3069 14679260 10.1001/jama.290.23.3066-b

[B63] Bridges CM Acarbose for patients with hypertension and impaired glucose tolerance JAMA 2003 290 23 3066 3067 author reply 3067-3069 14679261 10.1001/jama.290.23.3066-c

[B64] Chiasson JL Josse RG Gomis R Hanefeld M Karasik A Laakso M Acarbose treatment and the risk of cardiovascular disease and hypertension in patients with impaired glucose tolerance: the STOP-NIDDM trial JAMA 2003 290 4 486 494 10.1001/jama.290.4.486 12876091

[B65] Hanefeld M Pistrosch F Koehler C Chiasson JL Conversion of IGT to type 2 diabetes mellitus is associated with incident cases of hypertension: a post-hoc analysis of the STOP-NIDDM trial J Hypertens 2012 30 7 1440 1443 10.1097/HJH.0b013e328354663c 22573126

[B66] Hanefeld M Cagatay M Petrowitsch T Neuser D Petzinna D Rupp M Acarbose reduces the risk for myocardial infarction in type 2 diabetic patients: meta-analysis of seven long-term studies Eur Heart J 2004 25 1 10 16 10.1016/S0195-668X(03)00468-8 14683737

[B67] Rosenthal JH HM Effects on Blood Pressure of the Alpha-Glucosidase Inhibitor Acarbose Compared With the Insulin Enhancer Glibenclamide in Patients With Hypertension and Type 2 Diabetes Mellitus Clin Drug Invest 2002 22 695 701 10.2165/00044011-200222100-00006

[B68] Fukushima T Asahina M Fujinuma Y Yamanaka Y Katagiri A Mori M Kuwabara S Role of intestinal peptides and the autonomic nervous system in postprandial hypotension in patients with multiple system atrophy J Neurol 2012 260 2 475 483 22983428 10.1007/s00415-012-6660-x

[B69] Gentilcore D Vanis L Wishart JM Rayner CK Horowitz M Jones KL The alpha (alpha)-glucosidase inhibitor, acarbose, attenuates the blood pressure and splanchnic blood flow responses to intraduodenal sucrose in older adults J Gerontol A Biol Sci Med Sci 2011 66 8 917 924 21628676 10.1093/gerona/glr086

[B70] Kado S Murakami T Aoki A Nagase T Katsura Y Noritake M Matsuoka T Nagata N Effect of acarbose on postprandial lipid metabolism in type 2 diabetes mellitus Diabetes Res Clin Pract 1998 41 1 49 55 10.1016/S0168-8227(98)00062-X 9768372

[B71] Buse JB Tan MH Prince MJ Erickson PP The effects of oral anti-hyperglycaemic medications on serum lipid profiles in patients with type 2 diabetes Diabetes Obes Metab 2004 6 2 133 156 10.1111/j.1462-8902.2004.00325.x 14746579

[B72] Chiasson JL Josse RG Gomis R Hanefeld M Karasik A Laakso M Acarbose for prevention of type 2 diabetes mellitus: the STOP-NIDDM randomised trial Lancet 2002 359 9323 2072 2077 10.1016/S0140-6736(02)08905-5 12086760

[B73] Monami M Vitale V Ambrosio ML Bartoli N Toffanello G Ragghianti B Monami F Marchionni N Mannucci E Effects on lipid profile of dipeptidyl peptidase 4 inhibitors, pioglitazone, acarbose, and sulfonylureas: meta-analysis of placebo-controlled trials Adv Ther 2012 29 9 736 746 10.1007/s12325-012-0045-5 22923161

[B74] Santilli F Formoso G Sbraccia P Averna M Miccoli R Di Fulvio P Ganci A Pulizzi N Lattanzio S Ciabattoni G Consoli A Lauro R Patrono C Davi G Postprandial hyperglycemia is a determinant of platelet activation in early type 2 diabetes mellitus J Thromb Haemost 2010 8 4 828 837 10.1111/j.1538-7836.2010.03742.x 20088941

[B75] Monnier L Mas E Ginet C Michel F Villon L Cristol JP Colette C Activation of oxidative stress by acute glucose fluctuations compared with sustained chronic hyperglycemia in patients with type 2 diabetes JAMA 2006 295 14 1681 1687 10.1001/jama.295.14.1681 16609090

[B76] Heine RJ Balkau B Ceriello A Del Prato S Horton ES Taskinen MR What does postprandial hyperglycaemia mean? Diabet Med 2004 21 3 208 213 10.1111/j.1464-5491.2004.01149.x 15008828

[B77] Standl E Schnell O Ceriello A Postprandial hyperglycemia and glycemic variability. Should we care? Diabetes Care 2011 34 Suppl 2 120 127 10.2337/dc11-s206 PMC3632148 21525442

[B78] Coutinho M Gerstein HC Wang Y Yusuf S The relationship between glucose and incident cardiovascular events. A metaregression analysis of published data from 20 studies of 95,783 individuals followed for 12.4 years Diabetes Care 1999 22 2 233 240 10.2337/diacare.22.2.233 10333939

[B79] Nakagami T Hyperglycaemia and mortality from all causes and from cardiovascular disease in five populations of Asian origin Diabetologia 2004 47 3 385 394 10.1007/s00125-004-1334-6 14985967

[B80] Tominaga M Eguchi H Manaka H Igarashi K Kato T Sekikawa A Impaired glucose tolerance is a risk factor for cardiovascular disease, but not impaired fasting glucose. The Funagata Diabetes Study Diabetes Care 1999 22 6 920 924 10.2337/diacare.22.6.920 10372242

[B81] Borg R Kuenen JC Carstensen B Zheng H Nathan DM Heine RJ Nerup J Borch-Johnsen K Witte DR HbA (1) (c) and mean blood glucose show stronger associations with cardiovascular disease risk factors than do postprandial glycaemia or glucose variability in persons with diabetes: the A1C-Derived Average Glucose (ADAG) study Diabetologia 2011 54 1 69 72 10.1007/s00125-010-1918-2 20886203 PMC2995856

[B82] Barr EL Zimmet PZ Welborn TA Jolley D Magliano DJ Dunstan DW Cameron AJ Dwyer T Taylor HR Tonkin AM Wong TY McNeil J Shaw JE Risk of cardiovascular and all-cause mortality in individuals with diabetes mellitus, impaired fasting glucose, and impaired glucose tolerance: the Australian Diabetes, Obesity, and Lifestyle Study (AusDiab) Circulation 2007 116 2 151 157 10.1161/CIRCULATIONAHA.106.685628 17576864

[B83] Barr EL Boyko EJ Zimmet PZ Wolfe R Tonkin AM Shaw JE Continuous relationships between non-diabetic hyperglycaemia and both cardiovascular disease and all-cause mortality: the Australian Diabetes, Obesity, and Lifestyle (AusDiab) study Diabetologia 2009 52 3 415 424 10.1007/s00125-008-1246-y 19130039

[B84] Esposito K Ciotola M Carleo D Schisano B Sardelli L Di Tommaso D Misso L Saccomanno F Ceriello A Giugliano D Post-meal glucose peaks at home associate with carotid intima-media thickness in type 2 diabetes J Clin Endocrinol Metab 2008 93 4 1345 1350 10.1210/jc.2007-2000 18198229

[B85] Hanefeld M Fischer S Julius U Schulze J Schwanebeck U Schmechel H Ziegelasch HJ Lindner J Risk factors for myocardial infarction and death in newly detected NIDDM: the Diabetes Intervention Study, 11-year follow-up Diabetologia 1996 39 12 1577 1583 10.1007/s001250050617 8960845

[B86] Cavalot F Petrelli A Traversa M Bonomo K Fiora E Conti M Anfossi G Costa G Trovati M Postprandial blood glucose is a stronger predictor of cardiovascular events than fasting blood glucose in type 2 diabetes mellitus, particularly in women: lessons from the San Luigi Gonzaga Diabetes Study J Clin Endocrinol Metab 2006 91 3 813 819 10.1210/jc.2005-1005 16352690

[B87] Meier JJ Baller B Menge BA Gallwitz B Schmidt WE Nauck MA Excess glycaemic excursions after an oral glucose tolerance test compared with a mixed meal challenge and self-measured home glucose profiles: is the OGTT a valid predictor of postprandial hyperglycaemia and vice versa? Diabetes Obes Metab 2009 11 3 213 222 10.1111/j.1463-1326.2008.00922.x 18564177

[B88] Ceriello A Esposito K Piconi L Ihnat MA Thorpe JE Testa R Boemi M Giugliano D Oscillating glucose is more deleterious to endothelial function and oxidative stress than mean glucose in normal and type 2 diabetic patients Diabetes 2008 57 5 1349 1354 10.2337/db08-0063 18299315

[B89] Schisano B Tripathi G McGee K McTernan PG Ceriello A Glucose oscillations, more than constant high glucose, induce p53 activation and a metabolic memory in human endothelial cells Diabetologia 2011 54 5 1219 1226 10.1007/s00125-011-2049-0 21287141

[B90] Scognamiglio R Negut C de Kreutzenberg SV Tiengo A Avogaro A Effects of different insulin regimes on postprandial myocardial perfusion defects in type 2 diabetic patients Diabetes Care 2006 29 1 95 100 10.2337/diacare.29.01.06.dc05-0955 16373903

[B91] Esposito K Giugliano D Nappo F Marfella R Regression of carotid atherosclerosis by control of postprandial hyperglycemia in type 2 diabetes mellitus Circulation 2004 110 2 214 219 10.1161/01.CIR.0000134501.57864.66 15197140

[B92] Wascher TC Schmoelzer I Wiegratz A Stuehlinger M Mueller-Wieland D Kotzka J Enderle M Reduction of postchallenge hyperglycaemia prevents acute endothelial dysfunction in subjects with impaired glucose tolerance Eur J Clin Invest 2005 35 9 551 557 10.1111/j.1365-2362.2005.01550.x 16128861

[B93] Shimabukuro M Higa N Chinen I Yamakawa K Takasu N Effects of a single administration of acarbose on postprandial glucose excursion and endothelial dysfunction in type 2 diabetic patients: a randomized crossover study J Clin Endocrinol Metab 2006 91 3 837 842 10.1210/jc.2005-1566 16368744

[B94] Kato T Inoue T Node K Postprandial endothelial dysfunction in subjects with new-onset type 2 diabetes: an acarbose and nateglinide comparative study Cardiovasc Diabetol 2010 9 12 10.1186/1475-2840-9-12 20334663 PMC2861640

[B95] Bengel FM Abletshauser C Neverve J Schnell O Nekolla SG Standl E Schwaiger M Effects of nateglinide on myocardial microvascular reactivity in Type 2 diabetes mellitus–a randomized study using positron emission tomography Diabet Med 2005 22 2 158 163 10.1111/j.1464-5491.2004.01371.x 15660732

[B96] Nalysnyk L Hernandez-Medina M Krishnarajah G Glycaemic variability and complications in patients with diabetes mellitus: evidence from a systematic review of the literature Diabetes Obes Metab 2010 12 4 288 298 10.1111/j.1463-1326.2009.01160.x 20380649

[B97] Snell-Bergeon JK Roman R Rodbard D Garg S Maahs DM Schauer IE Bergman BC Kinney GL Rewers M Glycaemic variability is associated with coronary artery calcium in men with Type 1 diabetes: the Coronary Artery Calcification in Type 1 Diabetes study Diabet Med 2010 27 12 1436 1442 10.1111/j.1464-5491.2010.03127.x 21059097 PMC3052953

[B98] Muggeo M Zoppini G Bonora E Brun E Bonadonna RC Moghetti P Verlato G Fasting plasma glucose variability predicts 10-year survival of type 2 diabetic patients: the Verona Diabetes Study Diabetes Care 2000 23 1 45 50 10.2337/diacare.23.1.45 10857967

[B99] Lin SD Wang JS Hsu SR Sheu WH Tu ST Lee IT Su SL Lin SY Wang SY Hsieh MC The beneficial effect of alpha-glucosidase inhibitor on glucose variability compared with sulfonylurea in Taiwanese type 2 diabetic patients inadequately controlled with metformin: preliminary data J Diabetes Complications 2011 25 5 332 338 10.1016/j.jdiacomp.2011.06.004 21813293

[B100] Pistrosch F Schaper F Passauer J Koehler C Bornstein SR Hanefeld M Effects of the alpha glucosidase inhibitor acarbose on endothelial function after a mixed meal in newly diagnosed type 2 diabetes Horm Metab Res 2009 41 2 104 108 10.1055/s-0028-1103276 19061152

[B101] Kitano D Chiku M Li Y Okumura Y Fukamachi D Takayama T Hiro T Saito S Hirayama A Miglitol improves postprandial endothelial dysfunction in patients with acute coronary syndrome and new-onset postprandial hyperglycemia Cardiovasc Diabetol 2013 12 92 10.1186/1475-2840-12-92 23777506 PMC3691582

[B102] Ohkubo Y Kishikawa H Araki E Miyata T Isami S Motoyoshi S Kojima Y Furuyoshi N Shichiri M Intensive insulin therapy prevents the progression of diabetic microvascular complications in Japanese patients with non-insulin-dependent diabetes mellitus: a randomized prospective 6-year study Diabetes Res Clin Pract 1995 28 2 103 117 10.1016/0168-8227(95)01064-K 7587918

[B103] Kim DM Ahn CW Park JS Cha BS Lim SK Kim KR Lee HC Huh KB An implication of hypertriglyceridemia in the progression of diabetic nephropathy in metabolically obese, normal weight patients with type 2 diabetes mellitus in Korea Diabetes Res Clin Pract 2004 66 Suppl 1 S169 S172 15563971 10.1016/j.diabres.2004.07.011

[B104] Uzu T Yokoyama H Itoh H Koya D Nakagawa A Nishizawa M Maegawa H Yokomaku Y Araki S Abiko A Haneda M Elevated serum levels of interleukin-18 in patients with overt diabetic nephropathy: effects of miglitol Clin Exp Nephrol 2011 15 1 58 63 10.1007/s10157-010-0343-7 20824296

[B105] Creutzfeldt W Effects of the alpha-glucosidase inhibitor acarbose on the development of long-term complications in diabetic animals: pathophysiological and therapeutic implications Diabetes Metab Res Rev 1999 15 4 289 296 10.1002/(SICI)1520-7560(199907/08)15:4<289::AID-DMRR48>3.0.CO;2-V 10495478

[B106] Yamasaki Y Katakami N Hayaishi-Okano R Matsuhisa M Kajimoto Y Kosugi K Hatano M Hori M Alpha-Glucosidase inhibitor reduces the progression of carotid intima-media thickness Diabetes Res Clin Pract 2005 67 3 204 210 10.1016/j.diabres.2004.07.012 15713352

[B107] Hanefeld M Chiasson JL Koehler C Henkel E Schaper F Temelkova-Kurktschiev T Acarbose slows progression of intima-media thickness of the carotid arteries in subjects with impaired glucose tolerance Stroke 2004 35 5 1073 1078 10.1161/01.STR.0000125864.01546.f2 15073402

[B108] Koyasu M Ishii H Watarai M Takemoto K Inden Y Takeshita K Amano T Yoshikawa D Matsubara T Murohara T Impact of acarbose on carotid intima-media thickness in patients with newly diagnosed impaired glucose tolerance or mild type 2 diabetes mellitus: A one-year, prospective, randomized, open-label, parallel-group study in Japanese adults with established coronary artery disease Clin Ther 2010 32 9 1610 1617 10.1016/j.clinthera.2010.07.015 20974318

[B109] Oyama T Saiki A Endoh K Ban N Nagayama D Ohhira M Koide N Miyashita Y Shirai K Effect of acarbose, an alpha-glucosidase inhibitor, on serum lipoprotein lipase mass levels and common carotid artery intima-media thickness in type 2 diabetes mellitus treated by sulfonylurea J Atheroscler Thromb 2008 15 3 154 159 10.5551/jat.E549 18603822

[B110] Geng DF Jin DM Wu W Fang C Wang JF Effect of alpha-glucosidase inhibitors on the progression of carotid intima-media thickness: a meta-analysis of randomized controlled trials Atherosclerosis 2011 218 1 214 219 10.1016/j.atherosclerosis.2011.05.004 21640349

[B111] Patel YR Kirkman MS Considine RV Hannon TS Mather KJ Effect of acarbose to delay progression of carotid intima-media thickness in early diabetes Diabetes Metab Res Rev 2013 29 7 582 591 23908125 10.1002/dmrr.2433 PMC4062388

[B112] Ridker PM The JUPITER trial: results, controversies, and implications for prevention Circ Cardiovasc Qual Outcomes 2009 2 3 279 285 10.1161/CIRCOUTCOMES.109.868299 20031849

[B113] Rudofsky G Jr Reismann P Schiekofer S Petrov D von Eynatten M Humpert PM Isermann B Muller-Hoff C Thai TP Lichtenstein S Bartsch U Hamann A Nawroth P Bierhaus A Reduction of postprandial hyperglycemia in patients with type 2 diabetes reduces NF-kappaB activation in PBMCs Horm Metab Res 2004 36 9 630 638 10.1055/s-2004-825904 15486815

[B114] Lu JM Wang X Pan C Comparison of serum C-reactive protein level in different glucose tolerance subjects and the change in serum CRP level in IGT subjects with acarbose Chin J Endocrinol Metab 2003 19 254 256

[B115] Hanefeld M Schaper F Koehler C Bergmann S Ugocsai P Stelzer J Schmitz G Effect of acarbose on postmeal mononuclear blood cell response in patients with early type 2 diabetes: the AI (I) DA study Horm Metab Res 2009 41 2 132 136 10.1055/s-0028-1119407 19214923

[B116] Hasegawa G Kajiyama S Tanaka T Imai S Kozai H Fujinami A Ohta M Obayashi H Park H Nakano K Tanaka M Shiraishi E Fukui M Yoshikawa T Nakamura N The alpha-glucosidase inhibitor acarbose reduces the net electronegative charge of low-density lipoprotein in patients with newly diagnosed type 2 diabetes Clin Chim Acta 2008 390 1–2 110 114 18230353 10.1016/j.cca.2008.01.005

[B117] Miyamura M Schnell O Yamashita C Yoshioka T Matsumoto C Mori T Ukimura A Kitaura Y Matsumura Y Ishizaka N Hayashi T Effects of acarbose on the acceleration of postprandial hyperglycemia-induced pathological changes induced by intermittent hypoxia in lean mice J Pharmacol Sci 2010 114 1 32 40 10.1254/jphs.10014FP 20703014

[B118] Holman RR [Principal Investigator] Acarbose Cardiovascular Evaluation Trial (ACE) 2014 Available at http://clinicaltrials.gov/show/NCT00829660 (Accessed 19-02-2014)

